# Identification of loci governing soybean seed protein content *via* genome-wide association study and selective signature analyses

**DOI:** 10.3389/fpls.2022.1045953

**Published:** 2022-12-02

**Authors:** Hongmei Zhang, Guwen Zhang, Wei Zhang, Qiong Wang, Wenjing Xu, Xiaoqing Liu, Xiaoyan Cui, Xin Chen, Huatao Chen

**Affiliations:** ^1^ Institute of Industrial Crops, Jiangsu Academy of Agricultural Sciences, Nanjing Jiangsu, China; ^2^ Institute of Vegetables, Zhejiang Academy of Agricultural Sciences, Hangzhou Zhejiang, China; ^3^ College of Horticulture, Nanjing Agricultural University, Nanjing, Jiangsu, China

**Keywords:** protein content, GWAS, Selective signature analysis, candidate genes, soybean

## Abstract

Soybean [*Glycine max* (L.) Merr.] is an excellent source of protein. Understanding the genetic basis of protein content (PC) will accelerate breeding efforts to increase soybean quality. In the present study, a genome-wide association study (GWAS) was applied to detect quantitative trait loci (QTL) for PC in soybean using 264 re-sequenced soybean accessions and a high-quality single nucleotide polymorphism (SNP) map. Eleven QTL were identified as associated with PC. The QTL *qPC-14* was detected by GWAS in both environments and was shown to have undergone strong selection during soybean improvement. Fifteen candidate genes were identified in *qPC-14*, and three candidate genes showed differential expression between a high-PC and a low-PC variety during the seed development stage. The QTL identified here will be of significant use in molecular breeding efforts, and the candidate genes will play essential roles in exploring the mechanisms of protein biosynthesis.

## Introduction

Approximately 68% of the protein powder produced worldwide is derived from soybean [*Glycine max* (L.) Merr.]. Furthermore, meat producers have found soybean meal to be the preferred protein source for poultry and livestock. The demand for higher protein content (PC) over recent years has pushed breeders to develop soybean seeds with higher levels of protein (Patil et al., 2017). It is therefore important to understand the variation in soybean seed PC. Breeding high-protein soybean varieties is of great significance for increasing soybean protein yield. However, understanding of the mechanism(s) controlling variation in PC is limited.

In recent decades, 252 quantitative trait loci (QTL) on 20 chromosomes have been discovered for soybean PC and published in the Soybean Genetics and Genomics Database (https://www.soybase.org/). Biparental populations were used as the genetic background in the studies that uncovered 248 of those QTL. Diers et al. (1992) evaluated 60 F_2:3_ lines (A81-356022×PI 468916) with a restriction fragment length polymorphism (RFLP) linkage map of soybean containing 252 markers over 2,147 cM. They identified eight major QTL associated with PC in linkage groups A, C, and K, which explained 12-42% of the phenotypic variation. A major QTL for PC was detected on chromosome 14, which explained 12.4% of the phenotypic variation (Zhang et al., 2004). The resolution of QTL mapping *via* biparental populations was also successfully used to validate major QTL for PC (Wang et al., 2015; Warrington et al., 2015; Piyaporn et al., 2016). However, the higher confidence intervals and lower genetic variation in biparental populations cause challenges in integrating the results of linkage mapping into breeding programs; genome-wide association studies (GWAS) are thus preferred to study all recombination events that have occurred in the linkage disequilibrium (LD)-based evolutionary history of natural populations (Li et al., 2019; Zhang et al., 2021).

In association panels, characteristics such as LD, genetic diversity, marker density, and population structure affect the resolution and accuracy of QTL detected *via* GWAS (Sonah et al., 2015; Zhang et al., 2022). In recent years, GWAS have not only been applied to different populations to identify QTL associated with PC in soybean (Sonah et al., 2015; Zhang et al., 2017; Li et al., 2019; Zhang T et al., 2019), but have also been applied to analyze other complex quantitative traits such as oil content, salt stress tolerance, agronomic traits, and yield-related traits (Hwang et al., 2014; Fang et al., 2017; Zhang W et al., 2019; Pang et al., 2020). These findings have confirmed GWAS as a suitable approach for identifying novel QTL.

To identify novel components of the genetic architecture underlying PC, we here re-sequenced 264 soybean accessions and analyzed the genomes with a high-resolution single nucleotide polymorphism (SNP) map. In total, 11 QTL related to PC were identified. One QTL that was significantly associated with PC, *qPC-14*, was shown to have undergone selection during soybean improvement. Three candidate genes exhibiting differential expression between cultivars during the seed development stage may be involved in regulating soybean PC. The genes and SNPs identified here are expected to contribute to cultivation of high-protein soybean varieties through marker-assisted selection (MAS) programs.

## Materials and methods

### Plant materials and phenotypic measurements

There were 264 accessions in the soybean population: 52 landraces and 212 improved varieties (Zhang et al., 2021). All materials were planted in Sanya City, Hainan Province (109.70° E, 18.31°N) in 2020 (E1), and in Nanjing City, Jiangsu Province (118.68°E, 32.50°N) in 2021 (E2). The experimental plots utilized a randomized complete block design in one row with three replicates. The dimensions for single row seeding were 4 m length by 0.5 m width with 0.13 m spacing. Seeds from duplicate samples were pooled and dried in an oven to a constant weight.

For each replication, 20 g of seeds were ground with a 1095 Knifetec sample mill (FOSS Tecator, Denmark). All grains were sieved (0.25 mm pore size) into sample bottles. For each sample, 0.2 g was weighed out and placed in a 300 mL disboil tube. Protein content was then determined using the Kjeldahl protein analyzer (SKD-1800) according to Bremner (1960).

### Statistical analyses of phenotypic data

Descriptive statistics and *h* ^2^ were calculated in R (http://www.Rproject.org/). The formula for *h* ^2^ was as follows:


$$h^{2}=\sigma_{g}^{2}∕\left(\sigma_{g}^{2}+\sigma_{ge}^{2}∕n+\sigma^{2}∕nr\right)$$

where *σ* ^2^_g_ is the genotypic variance, *σ* ^2^_ge_ is the genotype by environmental interaction variance, *σ* ^2^ is the error variance, *n* is the number of environments, and *r* is the number of replicates. Bar graphs and line graphs were generated in Origin v8.0.

### Genotyping and GWAS

The germplasm resource population contained 264 accessions. The physical distance of the LD decay was estimated as the position at which *r^2^* decreased to half of its maximum value (~120 kb) (Zhang et al., 2021). A total of 2,597,425 SNPs were used in the GWAS (Zhang et al., 2021). MLMs were generated using the Genomic Association and Prediction Integrated Tool (‘GAPIT’) package in R. To reduce false positives and increase statistical accuracy (Lipka et al., 2012), -log_10_(*p*) > 5 was set as the threshold for a correlation to be considered significant.

### Selective sweep analyses

The VCFtools package (Danecek et al., 2011) was used to analyze the nucleotide diversity (π) and *F_ST_* between landraces and cultivars using a 10 kb step size and a 100 kb sliding window. The top 5% of *θ*_π_ ratios and *F_ST_* values were classified as putative selective regions and highly differentiated regions, respectively. The window intersection areas of *F_ST_* and *θ*_π_ ratio were designated as potential selective regions. *F_ST_* ≥ 0.204 and *θ*_π_ ratio< 0.545 were the thresholds used for landraces vs. cultivars in the selective sweep analysis.

### RT−PCR for candidate genes

Two soybean genotypes were used to examine differences in the expression of genes related to soybean PC accumulation during seed development *via* transcriptomics: NPS233 (high PC) and NPS301 (low PC). These two varieties exhibiting different genotypes and protein content, in which NPS233 with S14_73926-A and NPS301 with SNP S14_73926-G. Seed samples were collected from the two accessions at 14, 21, and 28 DAF, then immediately frozen in liquid nitrogen and stored at -80°C prior to further use.

Total RNA was extracted using an RNAsimple Total RNA Kit (TIANGEN, China). First-strand cDNA synthesis was conducted with a TaKaRa Primer Script RT reagent kit and gDNA Eraser. RT-PCR was performed on an ABI 7500 system (Applied Biosystems, USA) using SYBR Green Real-time Master Mix (Toyobo). Tubulin (GenBank accession number: AY907703) was used as the internal control for expression normalization.

## Results

### Phenotypic variation in PC

**Figure 1:**
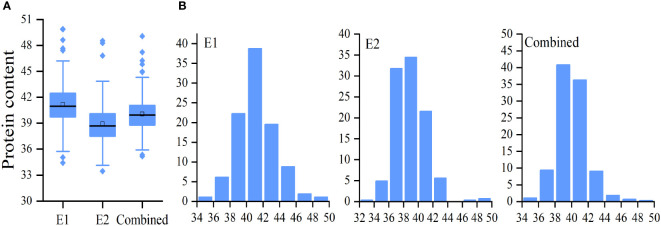
Distribution of soybean seed protein content (PC). Boxplot **(A)** and frequency distribution **(B)** of soybean seed PC in E1, E2, and combined environment, respectively.

A total of 264 soybean accessions were used in this study. Seed PC was measured in soybeans grown in 2020 (E1) and 2021 (E1) (**Table 1**). In addition, the mean value of E1 and E2, named as Combined, was used for data analysis. It was shown that differences in PC in the natural population were significant in E1,E2, and Combined (**Figure 1A** and **Table 1**). The range of soybean PC in the natural populations were 34.42% to 39.89% in E1, 33.46% to 48.53% in E2, and 35.18% and 49.07%, (**Table 1** and **Table S1**). The average PC in the natural population in Sanya was 41.17%, which was significantly higher than that of Nanjing (38.91%), indicating that PC was easily influenced by the growth environment. PC was normally distributed in the associated populations (**Figure 1B**), with extensive variation in trait phenotypes (**Table 1**). The 66.43% generalized heritability (*H^2^*) for PC indicated that genetic factors played a crucial role in soybean protein accumulation.

**Table 1:** Descriptive statistics for soybean protein content of the natural population.

En^a^	Min (%)	Max (%)	Range^b^(%)	Mean ± SD^c^	CV^d^(%)	*H^2 e^* (%)
E1	34.42	49.89	15.47	41.17 ± 2.42	5.89	66.43
E2	33.46	48.53	15.07	38.91 ± 2.10	5.4	
Combined	35.18	49.07	13.89	40.03 ± 1.95	4.87	

a E1, Sanya in 2020; E2, Nanjing in 2021, Combined, the mean value of E1 and E2. b Range, difference between maximum and minimum value. c Mean ± SD, mean ± standard deviation. d CV, coefficient of variation. e H2, broad-sense heritability.

### GWAS for PC in a natural population

**Figure 2:**
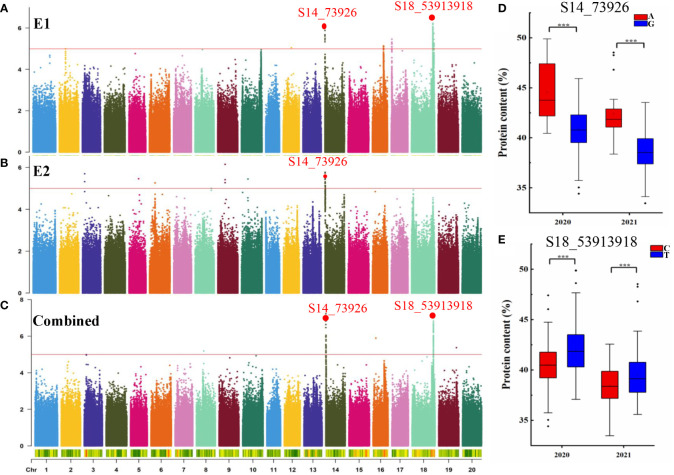
GWAS for seed protein content (PC). Manhattan plot for seed PC in E1 **(A)**, E2 **(B)**, and combined **(C)**. The red line indicates the significance threshold at -log_10_(*p*) = 5. **(D)** Boxplots for seed PC based on the S14_73926-A/G alleles in 2020 and 2021. **(E)** Boxplots for seed PC based on the S18_53913918-G/T alleles in 2020 and 2021. ***p < 0.001.

GWAS was conducted to detect SNPs associated with PC across two environments using 2,597,425 SNPs reported in a previous study (Zhang et al., 2021). To minimize false positives, a mixed linear model (MLM) was applied. The Manhattan plots for the GWAS results are shown in **Figures 2A–C**. PC was observed to be significantly correlated with 159 SNPs at a threshold of -log_10_(*p*) > 5 (**Table S2**). The SNPs were distributed across 11 chromosomes in E1, E2, and combined environment (**Figures 2A–C** and **Table 2**) and were responsible for phenotypic variation from 5.48 to 9.20% (**Table S2**). Among the significant SNPs, 30 were located on Chr.14; one of these, *qPC-14-1*, was represented by a peak at SNP S14_73926 and was detected in both 2020, 2021, and combined environment (*p* = 8.56×10^-7^, and 1.86×10^-6^, 5.93×10^-8^ respectively). *qPC-18-1*, located on Chr. 18, contained 99 SNPs; it was seen as a peak at SNP S18_53913918 and explained 9.20% of the total phenotypic variation in 2021 and combined environment. Moreover, we observed that soybean accessions carrying the S14_73926-A allele exhibited significantly higher average PC than those carrying the S14_73926-G allele (**Figure 2D**). Furthermore, soybean varieties carrying the S18_53913918-C allele showed significantly lower average PC than those carrying the S18_53913918-T allele (**Figure 2E**). In summary, GWAS yielded several loci that could explain the genetic variation responsible for differences in PC between genotypes.

**Table 2:** Loci and SNPs significantly associated with protein content, predicted candidate genes and previously reported QTL for protein content at similar genome regions.

QTL	Env^a^	Chr^b^	Position^c^	Lead SNP	−log_10_(*p*)	*R* ^2^(%)^d^	Known QTL
*qPC-3*	E2	Gm3	5232779	S03_5232779	5.70	6.38	Seed protein 4-9 (Lee et al., 1996); Seed protein 36-36 (Mao et al., 2013)
*qPC-5*	E2	Gm5	24270611	S05_24270611	5.46	6.07	Seed protein 36-1 (Mao et al., 2013);
*qPC-6*	E2	Gm6	13847565	S06_13847565	5.26	5.81	–
*qPC-8*	E2	Gm8	40497729	S08_40497729	5.01	5.48	–
*qPC-9*	E2	Gm9	18127054	S09_18127054	6.15	6.97	–
*qPC-10*	E2	Gm10	16047138	S10_16047138	5.45	6.05	Seed protein 36-40 (Mao et al., 2013)
*qPC-14*	E1,E2, combined	Gm14	73926	S14_73926	6.07	8.45	Seed protein 4-g4 (Sonah et al., 2015)
*qPC-16-1*	E1	Gm16	27931926	S16_27931926	5.14	6.97	–
*qPC-16-2*	E2	Gm16	6800169	S16_6800169	6.85	7.90	–
*qPC-17*	E1	Gm17	1545686	S17_1545686	5.46	7.47	–
*qPC-18*	E1, combined	Gm18	53913918	S18_53913918	6.52	9.20	Seed protein 1-8 (Diers et al., 1992),

a Environment.

b Chromosome.

c Most significant SNP position.

d The proportion of phenotypic variance explained by each QTL.

E1 and E2 represent two environments, 2020-year, and 2021-year, respectively.

Combined means the average value of E1 and E2.

### *qPC-14* is an improvement-selective QTL

**Figure 3:**
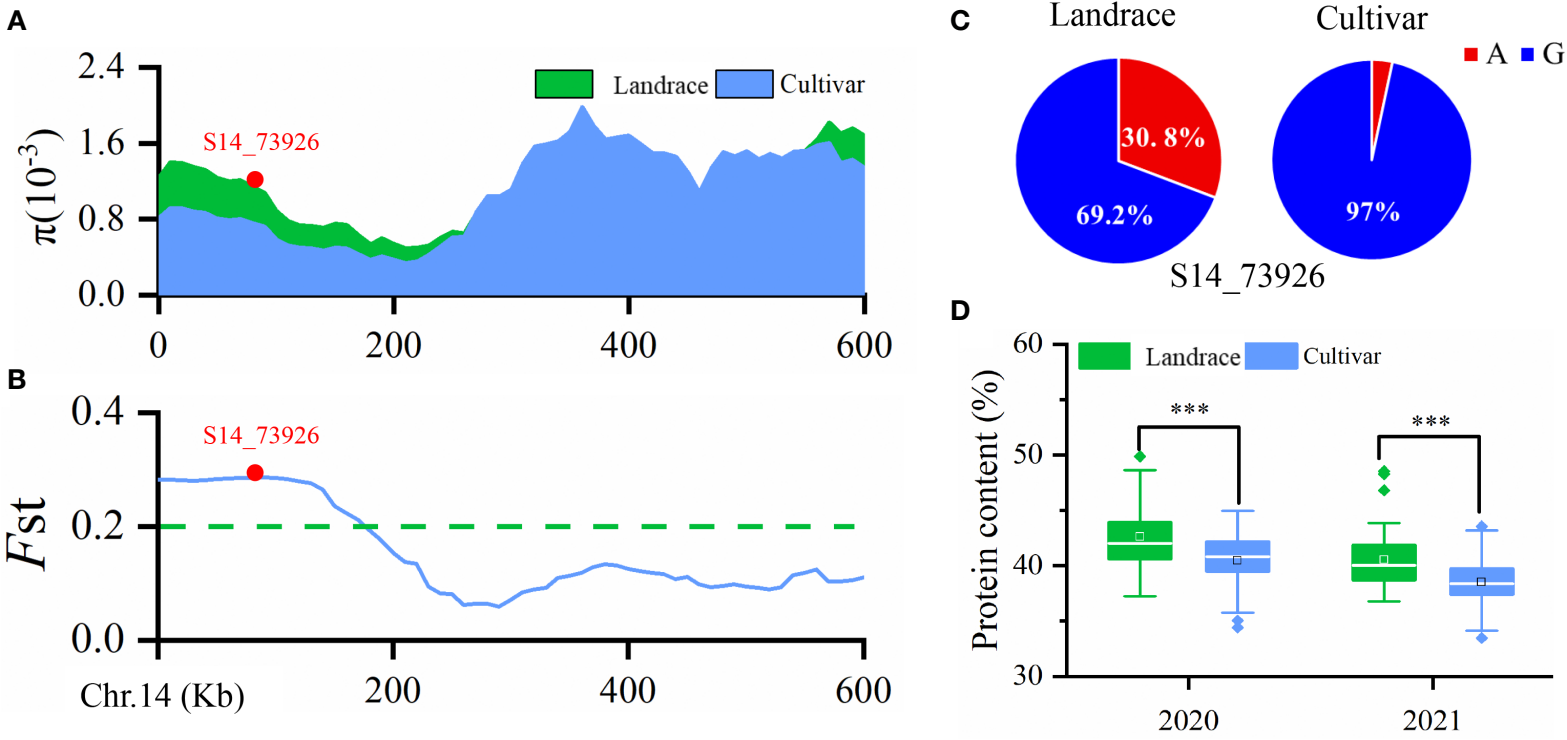
**(A)** Nucleotide diversity (π) values in landraces and improved cultivars. **(B)** Fixation index (*F*_ST_) plot of landraces and cultivars in the 600-kb genomic regions surrounding the lead SNP, S14_73926, in *qPC-14-1.* **(C)** Allele frequencies of S14_73926 in landraces and cultivars. A and G are the two different alleles for the SNP S14_73926. **(D)** Boxplot of soybean seed protein content (PC) in landraces and cultivars grown in 2020 and 2021. ***p < 0.001.

Wild soybean often has higher seed PC than landraces or improved cultivars, suggesting that seed PC has been under selection during soybean domestication and varietal improvement. Our previous study detected 39 improvement-selective regions for seed PC in a natural population containing 52 landraces and 212 cultivars (Zhang et al., 2021). In the present study, *qPC-14*, represented by SNP S14_73926, was located within a selective sweep region ~270 kb in size (Chr14: 0–270,000) as inferred by the fixation index (*F*_ST_) (≥ 0.204) (**Figure 3A**) and *θ*_π_ ratio (< 0.545) (**Figure 3B**). The data suggested that this GWAS signal, which was significantly associated with seed PC, had undergone strong selection during soybean improvement. Interestingly, we also observed that the frequency of the S14_73926-A allele was 30.8% in landraces, significantly higher than the 3.0% frequency in cultivars (**Figure 3C**). This difference was associated with a higher average seed PC in landraces (42.61% and 40.54%) than in cultivars (40.83% and 38.51%) (**Figure 3D**). Taken together, these results indicated that we identified a critical QTL for soybean PC through GWAS in the present study.

### Candidate genes for PC

**Figure 4:**
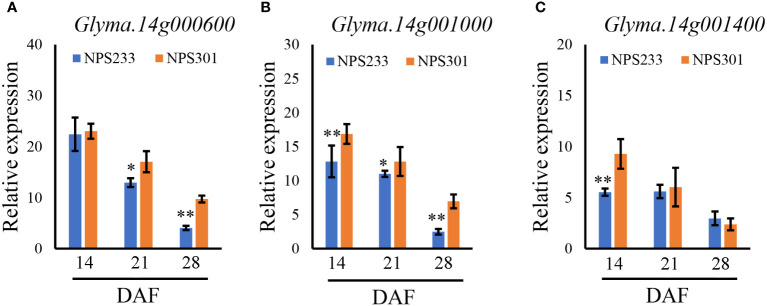
Relative expression patterns of *Glyma.14g000600* **(A)**, *Glyma.14g001000* **(B)**, and *Glyma.14g001400* **(C)** during seed development in the accessions NPS233 and NPS301. Seeds were sampled at 14, 21, and 28 days after flowering (DAF). *p< 0.05, **p< 0.01.

To identify candidate genes, we studied the 120 kb region flanking a significant representative SNP (S14_73926) based on the LD decay distance reported in a previous study (Zhang et al., 2021). Fifteen candidate genes were predicted in this region (**Table S3**). To determine which genes affected seed PC, quantitative reverse transcription (qRT)-PCR was conducted to analyze spatiotemporal expression patterns of the 15 candidate genes during seed development. Samples were collected from two soybean lines, NPS233 (with SNP S14_73926-A, a high protein variety) and NPS301 (with SNP S14_73926-G, a low protein variety) at 14, 21, and 28 d after flowering (DAF). The expression levels of two genes was too lowly to be detected (*Glyma.14g000100*, *Glyma.14g000200*, and *Glyma.14G000900*), and nine showed no significant differences in expression levels between NPS233 and NPS301 (*Glyma.14G000300*, *Glyma.14G000400*, *Glyma.14G000500*, *Glyma.14G000700*, *Glyma.14G000800*, *Glyma.14G001100*, *Glyma.14G001200*, *Glyma.14G001300*, and *Glyma.14G001500*). At 14 DAF, *Glyma.14G000600* was expressed at low levels with no significant difference between NPS233 and NPS301; however, this gene was then strongly downregulated in NPS233 compared to NPS301 (**Figure 4A**). Relative expression of *Glyma.14G001000* was significantly lower in NPS233 than in NPS301 during the seed development stage (**Figure 4B**). In addition, *Glyma.14G001400* was more highly expressed in NPS301 than in NPS233 at 14 DAF, but there was no difference in expression at 21 or 28 DAF (**Figure 4B**).

## Discussion

The goal of the present study was to identify SNPs and candidate genes significantly associated with soybean PC variation. A natural soybean population was re-sequenced and planted in two different geographical locations (Sanya in 2020 and Nanjing in 2021), and the phenotypes used to conduct GWAS for PC. It has previously been reported that soybean varieties planted at high latitudes exhibit lower PC than those planted at low latitudes. In addition, soybean PC is known to be affected by environmental factors, such as day length, temperature, and moisture levels (Song et al., 2016; Patil et al., 2017). Here, the average PC of a natural population plated in Sanya was significantly higher than the same population planted in Nanjing (**Table 1**). This suggests that combined climatic factors caused significant changes in PC and thus resulted in extensive phenotypic variation in the natural populations used for GWAS.

Over the past two decades, a great deal of research has been published related to genetic dissection of soybean PC. More than 200 QTL across 20 chromosomes have been identified as associated with seed PC (SoyBase, http://www.soybase.org/). A QTL related to seed PC and oil content has been located on Chr. 20 (Diers et al., 1992; Hwang et al., 2014; Warrington et al., 2015), and it has received extensive attention because of its high additive effects and stability (Diers et al., 1992; Wang et al., 2015; Li et al., 2019; Samanfar et al., 2019; Zhang W et al., 2019; Li et al., 2021; Goettel et al., 2022). *Glyma.20G085100* (*POWR1*) was recently reported as a causative gene of chr20 QTL; a transposable element insertion in its conserved CCT domain produced seeds with increased oil content and weight and decreased PC (Goettel et al., 2022). However, few QTL or genes related to PC have been applied in breeding due to limitations such as insignificant and unstable effects on phenotypes, negative correlations with seed oil and seed yield, and inconsistencies between growing environments (Wang et al., 2015; Goettel et al., 2022). It is therefore imperative to identify QTL with consistent and large effects on PC. In this study, to detect significant SNPs related to PC, a high-density map from a previous study (Zhang et al., 2021) was used. This map contained 2,597,425 SNPs, corresponding to ~2.6 SNP markers per kb, improving the precision of GWAS for studying complex traits. Eleven QTL for PC were detected *via* GWAS with an MLM method. Five were close to or overlapping with regions reported in previous studies, whereas six are reported here for the first time (**Table 2**). The QTL *qPC-3* identified here was located within three other QTL: *seed protein 4-9* (Lee et al., 1996), *seed protein 36-36*, and *seed protein 36-40* (Mao et al., 2013). *qPC-5* and *qPC-10* were each located within a previously mapped QTL, *seed protein 36-1* and *seed protein 36-40*, respectively (Mao et al., 2013). Although *qPC-18* was found to be associated with PC in 2020 and the SNP S18_53913918 accounted for 9.20% of the phenotypic variation, it also overlapped with a previously reported QTL (Diers et al., 1992), *seed protein 1-8* on Chr.18. *qPC-14* was significantly associated with PC in both 2020 and 2021 (**Table S2**). A previous GWAS showed that some SNP loci in this region regulated PC (Sonah et al., 2015). These results suggested that there are genes in these regions that regulate seed PC; future work should aim to identify candidate genes in these regions.

In China, demand for soybean has increased rapidly, and 80% of soybeans consumed were imported from Brazil and the United States of America in recent years. This trend has caused many breeders to prioritize high seed yield in recent years. However, there is a negative correlation between seed yield and PC (Rotundo et al., 2009; Patil et al., 2017), which prevents simultaneous increases in seed PC and yield. The breeding objective of high yield may result in development of more improved varieties with higher seed yield and lower PC compared with landrace varieties. Indeed, we found that the average PC was much higher in landrace varieties than in cultivars (*p*< 0.01).

Wild soybean is known to have much higher seed PC than landraces and cultivars (Hwang et al., 2014; Leamy et al., 2017; Li et al., 2019). This suggests that seed PC has undergone selection during domestication and improvement (Goettel et al., 2022). Domestication and improvement always resulted in selective sweeps, and significantly reduced nucleotide diversity in some regions of the genome (Hufford et al., 2012; Zhou et al., 2015; Zhang et al., 2021).Recently, a domestication gene, *POWR1*, was reported to regulate soybean protein content likely through controlling seed nutrient transport and lipid metabolism genes (Goettel et al., 2022). In the present study, to detect improvement-selection signals using soybean varieties collected from China, we scanned genomic regions with extreme allele frequency differences as described in our previous study (Zhang et al., 2021). An improvement-selection region was identified on Chr.14, which was overlapped with QTL *qPC*-*14* identified in this study. These results indicated that this QTL may have undergone selection during soybean improvement and was therefore a strong candidate gene for PC.

## Conclusions

Using a GWAS approach, 11 QTL were determined to be associated with PC in soybean. A reproducible and significant QTL, *qPC-14*, overlapped with a selective-sweep region on Chr.14, demonstrating that this QTL had undergone selection during soybean improvement. Three candidate genes showed differential expression patterns between the high PC accession NPS233 and the low PC accession NPS301 during seed development, and these genes may therefore regulate PC in soybean. The results presented here contribute to an improved understanding of the mechanisms that regulate PC in soybean seeds.

## Data availability statement

The original contributions presented in the study are included in the article/**Supplementary Material**. Further inquiries can be directed to the corresponding author.

## Author contributions

Conceptualization, HC. Formal analysis, HZ, WZ, and QW. Data curation, HZ and HC. Methodology, WZ, QW and WX. Investigation, HZ, and WX. Resources, HC and GZ. Funding acquisition, TH. Writing—original draft, HZ, and WZ. Writing—review and editing, HC, GZ, XL, XCu and XCh. All authors contributed to the article and approved the submitted version.

## Funding

This study was supported by National Key Research and Development Program of China (2018YFE0112200), the Key R&D project of Jiangsu Province (BE2019376), the open competition project of seed industry revitalization of Jiangsu Province (JBGS[2021]060), Jiangsu Agricultural Science and Technology Innovation Fund (CX(22)5002), the Natural Science Foundation of China (32001455), and the Natural Science Foundation of Jiangsu Province (BK20210154).

## Conflict of interest

The authors declare that the research was conducted in the absence of any commercial or financial relationships that could be construed as a potential conflict of interest.

## Publisher’s note

All claims expressed in this article are solely those of the authors and do not necessarily represent those of their affiliated organizations, or those of the publisher, the editors and the reviewers. Any product that may be evaluated in this article, or claim that may be made by its manufacturer, is not guaranteed or endorsed by the publisher.

## References

[B1] Bremner J. M. (1960). Determination of nitrogen in soil by the kjeldahl method. J. Agric. Sci. 55, 11–33. doi: 10.1017/S0021859600021572

[B2] Danecek P. Auton A. Abecasis G. Albers C. A. Banks E. DePristo M. A.. (2011). The variant call format and VCFtools. Bioinf. (Oxford England) 27, 2156–2158. doi: 10.1093/bioinformatics/btr330 PMC3137218 21653522

[B3] Diers B. W. Keim P. Fehr W. R. Shoemaker R. C. (1992). RFLP analysis of soybean seed protein and oil content. Theor. Appl. Genet. 83, 608–612. doi: 10.1007/BF00226905 24202678

[B4] Fang C. Ma Y. Wu S. Liu Z. Wang Z. Yang R.. (2017). Genome-wide association studies dissect the genetic networks underlying agronomical traits in soybean. Genome Biol. 18, 161. doi: 10.1186/s13059-017-1289-9 28838319 PMC5571659

[B5] Goettel W. Zhang H. Li Y. Qiao Z. Jiang H. Hou D.. (2022). POWR1 is a domestication gene pleiotropically regulating seed quality and yield in soybean. Nat. Commun. 13, 3051. doi: 10.1038/s41467-022-30314-7 35650185 PMC9160092

[B6] Hufford M. B. Xu X. van Heerwaarden J. Pyhäjärvi T. Chia J. Cartwright R. A.. (2012). Comparative population genomics of maize domestication and improvement. Nat. Genet. 44, 808–811. doi: 10.1038/ng.2309 22660546 PMC5531767

[B7] Hwang E. Song Q. Jia G. Specht J. E. Hyten D. L. Costa J.. (2014). A genome-wide association study of seed protein and oil content in soybean. BMC Genomics 15, 1. doi: 10.1186/1471-2164-15-1 24382143 PMC3890527

[B8] Leamy L. J. Zhang H. Li C. Chen C. Y. Song B. (2017). A genome-wide association study of seed composition traits in wild soybean (Glycine soja). BMC Genomics 18, 18. doi: 10.1186/s12864-016-3397-4 28056769 PMC5217241

[B9] Lee S. H. Bailey M. A. Mian M. A. R. Carter T. E. Shipe E. R. Ashley D. A.. (1996). RFLP loci associated with soybean seed protein and oil content across populations and locations. Theor. Appl. Genet. 93, 649–657. doi: 10.1007/BF00224058 24162390

[B10] Lipka A. E. Tian F. Wang Q. Peiffer J. Li M. Bradbury P. J.. (2012). GAPIT: genome association and prediction integrated tool. Bioinformatics 28, 2397–2399. doi: 10.1093/bioinformatics/bts444 22796960

[B11] Li X. Wang P. Zhang K. Liu S. Qi Z. Fang Y.. (2021). Fine mapping QTL and mining genes for protein content in soybean by the combination of linkage and association analysis. Theor. Appl. Genet. 134, 1095–1122. doi: 10.1007/s00122-020-03756-0 33420806

[B12] Li D. Zhao X. Han Y. Li W. Xie F. (2019). Genome-wide association mapping for seed protein and oil contents using a large panel of soybean accessions. Genomics 111, 90–95. doi: 10.1016/j.ygeno.2018.01.004 29325965

[B13] Mao T. Jiang Z. Han Y. Teng W. Zhao X. Li W.. (2013). Identification of quantitative trait loci underlying seed protein and oil contents of soybean across multi-genetic backgrounds and environments. Plant Breed. 6, 630–641. doi: 10.1111/pbr.12091

[B14] Pang Y. Liu C. Wang D. St. Amand P. Bernardo A. Li W.. (2020). High-resolution genome-wide association study identifies genomic regions and candidate genes for important agronomic traits in wheat. Mol. Plant 13, 1311–1327. doi: 10.1016/j.molp.2020.07.008 32702458

[B15] Patil G. Mian R. Vuong T. Pantalone V. Song Q. Chen P.. (2017). Molecular mapping and genomics of soybean seed protein: a review and perspective for the future. Theor. Appl. Genet. 130, 1975–1991. doi: 10.1007/s00122-017-2955-8 28801731 PMC5606949

[B16] Piyaporn P. Watcharin S. Hyten D. L. Qijian S. Cregan P. B. Graef G. L.. (2016). Multi-population selective genotyping to identify soybean [*Glycine max* (L.) merr.] seed protein and oil QTLs. G3 (Bethesda Md.) 6, 1635–1648. doi: 10.1534/g3.116.027656 27172185 PMC4889660

[B17] Rotundo J. L. Borrás L. Westgate M. E. Orf J. H. (2009). Relationship between assimilate supply per seed during seed filling and soybean seed composition. Field Crop Res. 112, 90–96. doi: 10.1016/j.fcr.2009.02.004

[B18] Samanfar B. Cober E. R. Charette M. Tan L. H. Bekele W. A. Morrison M. J.. (2019). Genetic analysis of high protein content in ‘AC proteus’related soybean populations using SSR, SNP, DArT and DArTseq markers. Sci. Rep-Uk 9, 19657. doi: 10.1038/s41598-019-55862-9 PMC6928212 31873115

[B19] Sonah H. O'Donoughue L. Cober E. Rajcan I. Belzile F. (2015). Identification of loci governing eight agronomic traits using a GBS-GWAS approach and validation by QTL mapping in soya bean. Plant Biotechnol. J. 13, 211–221. doi: 10.1111/pbi.12249 25213593

[B20] Song W. Yang R. Wu T. Wu C. Sun S. Zhang S.. (2016). Analyzing the effects of climate factors on soybean protein, oil contents, and composition by extensive and high-density sampling in China. J. Agr Food Chem. 64, 4121–4130. doi: 10.1021/acs.jafc.6b00008 27022763

[B21] Wang J. Chen P. Wang D. Shannon G. Zeng A. Orazaly M.. (2015). Identification and mapping of stable QTL for protein content in soybean seeds. Mol. Breed. 35, 92. doi: 10.1007/s11032-015-0285-6

[B22] Warrington C. V. Abdel-Haleem H. Hyten D. L. Cregan P. B. Orf J. H. Killam A. S.. (2015). QTL for seed protein and amino acids in the benning × danbaekkong soybean population. Theor. Appl. Genet. 128, 839–850. doi: 10.1007/s00122-015-2474-4 25673144

[B23] Zhang W. Liao X. Cui Y. Ma W. Zhang X. Du H.. (2019). A cation diffusion facilitator, GmCDF1, negatively regulates salt tolerance in soybean. PloS Genet. 15, e1007798. doi: 10.1371/journal.pgen.1007798 30615606 PMC6336350

[B24] Zhang D. Lü H. Chu S. Zhang H. Zhang H. Yang Y.. (2017). The genetic architecture of water-soluble protein content and its genetic relationship to total protein content in soybean. Sci. Rep-Uk 7, 5053. doi: 10.1038/s41598-017-04685-7 PMC5506034 28698580

[B25] Zhang W. K. Wang Y. J. Luo G. Z. Zhang J. S. He C. Y. Wu X. L.. (2004). QTL mapping of ten agronomic traits on the soybean (*Glycine max* l. merr.) genetic map and their association with EST markers. Theor. Appl. Genet. 108, 1131–1139. doi: 10.1007/s00122-003-1527-2 15067400

[B26] Zhang T. Wu T. Wang L. Jiang B. Zhen C. Yuan S.. (2019). A combined linkage and gwas analysis identifies QTLs linked to soybean seed protein and oil content. Int. J. Mol. Sci. 20, 5915. doi: 10.3390/ijms20235915 31775326 PMC6928826

[B27] Zhang W. Xu W. Li S. Zhang H. Liu X. Cui X.. (2022). GmAOC4 modulates seed germination by regulating JA biosynthesis in soybean. Theor. Appl. Genet. 135, 439–447. doi: 10.1007/s00122-021-03974-0 34674010

[B28] Zhang W. Xu W. Zhang H. Liu X. Cui X. Li S.. (2021). Comparative selective signature analysis and high-resolution GWAS reveal a new candidate gene controlling seed weight in soybean. Theor. Appl. Genet. 134, 1329–1341. doi: 10.1007/s00122-021-03774-6 33507340

[B29] Zhou Z. Jiang Y. Wang Z. Gou Z. Lyu J. Li W.. (2015). Resequencing 302 wild and cultivated accessions identifies genes related to domestication and improvement in soybean. Nat. Biotechnol. 33, 125–408. doi: 10.1038/nbt.3096 25643055

